# Supplementary material for: Patterns of Intron Gain and Loss in Fungi
Source: PLoS Biol. 2004 Nov 30;2(12):e422. doi: 10.1371/journal.pbio.0020422 (PMC532390; doi:10.1371/journal.pbio.0020422)
Supplement: Table S1 — Also available at http://genes.mit.edu/NielsenEtAl/. (4.3 MB ZIP). [file pbio.0020422.st001.zip › NielsenEtAl/html/1277.html]

AN4688.1.NCU02126.1.MG02540.1.FG08510.1


```
 CLUSTAL W (1.82) Multiple Sequence Alignments - Introns Inserted


Sequence 1: NCU02126.1	482 aa
Sequence 2: FG08510.1	426 aa
Sequence 3: AN4688.1	431 aa
Sequence 4: MG02540.1	470 aa
Alignment Length: 489 aa
Number Identitical Residues: 252 aa
Alignment Score (without introns) 11103


MG02540.1 	MASMRAVATLARPAAATLRP--QTLAALIPSSASASTTTSSSAINTSTTPSSRLRRSIP-
NCU02126.1	MSTQGLSRALARSTTTKSIPRIPTGISRVPRQSRLHSTYTATTTTTTTTTTTTTRRPSPT
FG08510.1 	MASVRTLRSLAR--------------PVVARQWR--------------------------
AN4688.1  	MATIRLPLLIRR--------------SLPTRAP---------------------------
          	*::      : *              .  .                              

MG02540.1 	---SRRQISTTAPRASSRAPKPKHPAGFVAPDQAALDELRASVQEFVR~AEIPEEVAART
NCU02126.1	TVSPLLTLNHIQTQRRNASHHNKHPAGFTPPSPTDLAELRERVQEFVR~RSLPETLAAET
FG08510.1 	---PAVTLPAIQSR----LHSSKHPKGFEAPSNEELDGLRERVQEFTR~REITEEVAAKT
AN4688.1  	---ARFTRPLIPCVR--TLATTKHPRGFVPPTEDDLLELRESVQEFTK1REIPEEVAART
          	   .                  *** ** .*    *  **  ****.:  .:.* :**.*

MG02540.1 	DKENEFPADMWGKLGDMG~LLGVAAEESVGGLAMGYQAHCIVMEELSRASA~SIALSYAA
NCU02126.1	DKSNAFPPHMWQALGDAG~LLGITADEAYGGLSMGYQAHCVVMEELSRASG~SIALSYAA
FG08510.1 	DKTNAFPAEMWQKLGEAG~FLGITADEDVGGLAMGYQAHIIVMEELSRASG~SIGLSYAA
AN4688.1  	DAQNEFPAEMWKKLGNAG2FLGVTADEEYGGLGMGYQAHCVVMEEISRASG1SIALSYAA
          	*  * **..**  **: * :**::*:*  ***.****** :****:****. **.*****

MG02540.1 	HSQLCVNQLQLNGNPEQKQRFMPDLIAGRKVGALAMSESGSGSDVVSMRTRATRTADGSG
NCU02126.1	HSQLCVNQLSLNGNAEQKEKYLPGLISGEKVGALAMSESGSGSDVVSMRT-TAKKVEGGG
FG08510.1 	HSQLCVNQLQLNGSPEQKQKYLPGLIAGTSVGALAMSESGAGSDVVSMRT-TAKAVDGG-
AN4688.1  	HSQLCVNQLSLNGTPEQKARFLPGLLSGEKIGALAMSEHSAGSDVVSMKT-SAKEVDGG-
          	*********.***..*** :::*.*::* .:******* .:*******:* ::: .:*. 

MG02540.1 	WLLNGSKMWITN~GPDADVIIVYAKSDTECQPSKGITAFIVER------QPGLQCLRKLD
NCU02126.1	YKLNGSKMWITN~GPDADVIVVYAKTEPDAG-SKGITAFIVETKKDGVKAEGFECLRKLD
FG08510.1 	YVLNGSKMWITN~GPDADFIVVYAKTEPEKN-SKGITAFIVDT-----KTEGFSCARKLD
AN4688.1  	WVLNGTKMWITN0GPDADYIVVYAKTEPEKG-SKGITAFVVEK-----TFKGFSCARKLD
          	: ***:****** ***** *:****::.:   *******:*:      .  *:.* ****

MG02540.1 	KMGMRGSNTGELVLENVIVPDENVLGEVNAGVRVLMEGLDLERLVLSAGPLG~IMQAALD
NCU02126.1	KMGMRGSNTGELVFENVFVPEENVLGKVNGGVRVLMEGLDLERLVLSAGPLG~LMQAALD
FG08510.1 	KMGMRGSNTGELMFDGVFVPTENILGKVNGGVRVLMEGLDLERLVLSAGPLG~IMQAALD
AN4688.1  	KLGMRGSNTGELIFEDVFVPRENLLGEVNRGVKVLMEGLDLERLVLSAGPLG2IMQAALD
          	*:**********:::.*:** **:**:** **:******************* :******

MG02540.1 	VALPYAHARQQFGRPIAEFQLVQGRLADMYTKLQVSRAYTYATARRIDESG-----EIKT
NCU02126.1	VALPYAHSRKQFGTPIAHFQLLQGKLADMYTKLQASRAYTYATAKTVDEQG-----VIKT
FG08510.1 	VTLPFTHQRKQFGQPIAHNQLLQGKLADMYTKLQASRCYTYATAKAVDENG-----LIRT
AN4688.1  	LVLPYTHVRKQFGAPIAHNQLIQGKLADMHTKLAASRAYTYATARHIDSHASLGSAAIRT
          	:.**::* *:*** ***. **:**:****:*** .**.******: :*. .: .:: *:*

MG02540.1 	ADCAGAILYAAERATEVALDCIQVLGGMGYTEDMPASRLLRD2AKLYEIGAGTSEVRRMV
NCU02126.1	QDCAGAILYAAERATEVALDAIQILGGMGYMEEMPVSRIMRD~AKLYEIGAGTSEVRRMV
FG08510.1 	QDCAGAILYAAERATECTLDCIQLLGGMGYVEEMPASRLLRD2AKLYEIGAGTSEIRRMV
AN4688.1  	QDCAGAILYAAERATECALDAIQLMGGNGYINEIPAGRLLRD~AKLYEIGAGTSEIRRMV
          	 *************** :**.**::** ** :::*..*::** ************:****

MG02540.1 	IGRYFNREYSLNQ--
NCU02126.1	IGRVFNKEYAHLANN
FG08510.1 	IGRAFNKEYAQA---
AN4688.1  	IGRAFNKEYAQ----
          	*** **:**:
```
